# The impact of COVID-19 pandemic on patients with Huntington's disease and care-givers: A French survey

**DOI:** 10.1016/j.ensci.2024.100517

**Published:** 2024-07-20

**Authors:** Sara Meoni, Elena Moro

**Affiliations:** a Université Grenoble Alpes, Inserm, U1216, CHU Grenoble Alpes, Grenoble Institut of Neurosciences, 38000 Grenoble, France; b “Aldo Ravelli” Center for Nanotechnology and Neurostimulation, University of Milan, Milan, Italy

**Keywords:** COVID-19, Epidemic, Huntington's disease, Survey

## Abstract

Although the impact of the first wave of the COVID-19 pandemic on people with several neurological diseases has been largely investigated, little is available concerning people with Huntington's disease (HD).

The main objective of the study was to interview people with HD and their caregivers in the Auvergne-Rhone Alpes region, France.

The interview consisted of 16 items concerning general and medical information, and the impact of the first wave of COVID-19 pandemic on the medical care of people with HD and on their caregivers. The questionnaire was made available as online survey from October 1st, 2020 until November 15th, 2020.

Fifty-two subjects participated (13 men, 39 women, mean age of 47.3 ± 15.5 years). Almost half participants (48%) experienced a worsening of pre-existing symptoms, with new-onset symptoms in the 44% of cases. The most frequent worsening was reported in gait and balance issues (67%), fatigue (58%), anxiety (50%), and depression (50%). The 70.8% of participants reported an inappropriate overall care of HD due to long delays to access medical care (30%) and other health care teams (60%). More than half of the participants (54.2%) reported that the COVID-19 pandemic had a negative impact on their caregiver/family.

Our findings emphasize the negative impact of the first wave of COVID-19 pandemic on the healthcare of HD population and their caregivers. Not only some symptoms were aggravated, but new symptoms appeared during the pandemic. In the future, health policies should be considered to improve the care of patients with rare diseases such as HD.

## Highlights

• The impact of COVID-19 pandemic on Huntington's disease is largely unknown.
• Almost half participants (48%) experienced a worsening of pre-existing symptoms.
• A new-onset symptoms was observed in the 44% of cases.
• The 70.8% of participants reported an inappropriate overall care of the disease.
• More than half of participants reported a negative impact of pandemic on caregivers.

## 1 Introduction

The new coronavirus disease (COVID-19) pandemic has resulted in unique stressors also for people with neurological disorders, and both their caregivers and neurologists [1]. Indeed, the management of patients with several neurological diseases, such as stroke [2,3], multiple sclerosis (MS) [4] and Parkinson's disease (PD) [5] has been greatly impacted by the first pandemic wave. Health care systems world-wide have implemented several strategies to face the sanitary emergency [6]. During the ‘safer-at-home’ orders, in-person clinical visits were restricted due to concerns of COVID-19 transmission. In response to the restrictions on in-person appointments, many clinics began adoption of telehealth visits [7,8].

The care for people with Huntington's disease (HD) requires complex, highly specialized, multidisciplinary management [9]. For instance, different professionals including neurologists, psychiatrists, psychologists, physical and occupational therapists, speech language pathologists, genetic counselors and social workers collaborate to take care of people with HD seen in a multi-disciplinary clinic [10]. Thus, the inability to interact with all of these providers may negatively impact patient health as well as place additional burdens on their caregivers [11].

So far, some surveys have been conducted focusing on the overall impact of COVID-19 pandemic on several neurologic disorders [12], including epilepsy [13], PD [5,14], MS [15], most reporting significant issues in the delivery of health care and patients' fears for future pandemic waves.

To date, no data is available about the medical, personal and social impact of the first wave of COVID-19 pandemic in people with HD and their caregivers. In order to fill this gap, we conducted an online survey in the HD population in the Auvergne-Rhone Alpes region, France.

## 2 Material and methods

### 2.1 Participants

Any subject with HD and his/her caregiver living in the Auvergne-Rhone-Alpes department (France) could participate to the survey. The informed consent was acquired before validating the submission of the survey.

### 2.2 Survey structure and dissemination

The survey was structured in three main sections including 16 items: (a) general information (four items); (b) medical information about HD and COVID-19 disease in participants during the first wave of pandemic and in the three following months (six items); (c) impact of COVID-19 on overall standard care of patients with HD and its impact on caregivers (six items) (Table 1).

**Table 1:** The HD survey (English translation) on the impact of COVID-19 pandemic on patients with Huntington's disease and their caregivers in the Auvergne-Rhone Alpes region.

**Section I: General information**
1) Who is the person completing the survey? (The patient - The caregiver - Both)
2) How old are you? (Select the numbers of years)
3) Are you a man or a woman? (Man – Woman)
4) Where do you live? (At home alone – At home with family- Institution)
**Section II: Medical information about HD and COVID-19 disease**
1) Do you have any HD symptoms? (Yes – No)
2) How many years have you had HD symptoms? (Select the numbers of years)
3) Do you need help in daily life activities? (No help – Help for some activities – Completely dependent)
4) Did you have a diagnosis of COVID-19? (Yes – No) If Yes, how was the diagnosis confirmed? (Swab – Serological test - Both) If Yes, have you been hospitalized? (Yes – No) If Yes, how did the COVID-19 change your HD symptoms? (No change – Worsening of symptoms – New symptoms onset) If Yes, did you experience a social isolation? (Yes – No)
5) Did the global health crisis due to the first wave of COVID-19 modify your HD symptoms during the three following months? (Yes, I had a worsening of my usual symptoms – Yes, I had new symptoms- No change) If Yes, I had a worsening of my usual symptoms: which ones? (Select the text, more than one answer is possible) If Yes, I had new symptoms: which ones? (Select the text, more than one answer is possible)
6) Did you need to change your medical treatment during the first wave of pandemic and the three following months? (Yes, I needed to increase the daily dose and/or the frequency of one or more medications – Yes, I needed to add one or more new medications- No change)
**Section III: The impact of COVID-19 on overall standard care of patients with HD and on their caregivers**
1) Did you experience any problem in the standard care of your HD during the first pandemic and the three following months? (Yes – No) If Yes, which are the problems identified? (Select the text, more than one answer is possible)
2) During the pandemic, was the standard neurological follow-up realized? Yes, by in-person visits- Yes, by phone calls- Yes, by virtual meetings- Not realized at all (more than one answer is possible).
3) Did you need a psychological follow-up during and after the first pandemic? (Yes – No) If Yes, how was it possible? (By in-person visits- By phone calls- By virtual meetings- Not possible)
4) Did you need to increase or start a psychological follow-up during and after the pandemic? (Yes – No) If Yes, was it possible? (Yes – No)
5) Do you think that the difficulties in the management of your HD during and after the pandemic negatively impacted your caregiver(s) and/or your family? (Yes – No)
6) Do you think that the overall management of your HD was appropriate during and after the pandemic? (Yes – No) If No, how do you think it could be improved in the future? (Select the text, more than one answer is possible)

The online survey was in French language. The link was disseminated by the Auvergne-Rhone-Alpes subsection of the national French HD patients' Association (Association Huntington France, AHF) mailing list. Moreover, the mobile HD team (a regional team including a psychologist, an occupational therapist and a management coordinator, moving at HD patients' home as needed) helped to disseminate the link by its mailing list. The link was also diffused by the AHF online newsletter. Overall, the survey link was sent to 200 subjects.

The survey was available from October 1st, 2020 until November 15th, 2020.

### 2.3 Statistical analysis

Descriptive statistics (mean and standard deviation (SD)) for continuous variables and percentages for categorical variables were performed to conduct the data analysis.

Fisher test for development of COVID-19 versus worsening of preexisting symptoms or appearance of new symptoms, and perception of insufficient care versus worsening of preexisting symptoms or appearance of new symptoms was also performed. A *p* value <0.05 was considered significant.

## 3 Results

A total of 52 participants completed the survey.

### 3.1 General information

Most participants were women (75%), with a mean age of 47.3 years (SD 15.5).

In 50% of cases, they lived alone at home; 34% of participants lived with family and 16% were institutionalized. Most of them (75%) could complete the survey without the caregiver help.

### 3.2 Clinical information about HD and COVID-19

Most participants had symptomatic HD (92%), with a mean disease duration of 9.8 years (SD 6.8). Concerning the activities of daily living, most of them were still completely independent (46%), 38% of cases partially dependent and the remaining 16% completely dependent. The 8% of participants were asymptomatic HD mutation carriers.

Regarding the COVID-19 disease, 20% of participants was diagnosed with the infection, confirmed by PCR testing in all cases. None of the them needed hospitalization and, interestingly, none reported worsening of HD symptoms during the COVID-19 infection. A social stigma related to the infection was reported in 25% of cases.

Concerning the overall impact of COVID-19 on the HD symptoms during the first wave of the pandemic and the three following months, almost half participants (48%) experienced a worsening of one (30%) or more (70%) pre-existing symptoms, with one (40%) or more (60%) new-onset symptoms in 44% of cases. The worsening of pre-existing HD symptoms concerned most frequently gait and balance issues (67%), fatigue (58%), anxiety (50%), and depression (50%). The participants reported also speech and swallowing issues (42%), feeling stressed (33%), anger and irritability (33%), cognitive issues (25%), and changes in appetite (25%). Chorea (17%) and sleep disorders (17%) were less impacted.

The most frequent new-onset symptoms were anxiety (50%), depression (45%), gait and balance issues (40%).

Medical treatment adjustments (increase of the dose and/or of the frequency of medications) were needed in 25% of participants experiencing worsening of HD symptoms. Thirty percent of the participants with new-onset symptoms needed to start a new medication.

In 12.5% of cases, psychological follow-ups were increased in frequency or were started.

### 3.3 Impact of COVID-19 on overall standard care of people with HD and its impact on caregivers

Most participants (70.8%) reported an inappropriate overall care of HD during and three months following the first wave of the pandemic. The main reasons were long delays to access medical care (30%) and other health care teams (60%), with impossible access in some cases (10%). Twelve percent of participants had difficulties to get medications. Almost one third of cases (32%) suffered of social isolation.

Neurological standard care was maintained through regular in-person consultations (37%), phone calls with medical staff (20.8%) and virtual consultations (8%).

Regular psychological sessions were possible in person (33%), by phone calls (22%), virtual meetings (22%) or not possible at all (33%). Increasing or starting a psychologist follow-up was impossible in the 66% of cases.

Almost 30% of participants judged the new approaches (virtual consultations and phone calls) not adequate for the regular care of their disease.

More than half participants (54.2%) reported that the COVID-19 pandemic had a negative impact on their caregiver/family.

Concerning the improvement in management of people with HD during possible future waves of pandemic, the majority of the survey respondents suggested an easier and faster medical access (57%), and more availability of health professionals' teams for at-home care (42%).

Twenty-eight percent of participants also expressed the need of more information about the impact of the COVID-19 disease on HD. For the future, they hope the development of clinical research protocols for HD (14%) and of telemedicine as tools to add to in-person visits (7%).

No significant association was found between the development of COVID-19 and worsening of preexisting symptoms or appearance of new symptoms, or perception of insufficient care and worsening of preexisting symptoms or appearance of new symptoms (*p* < 0.05).

## 4 Discussion

Our survey investigated the global impact of the first wave of COVID-19 pandemic on the HD population in the French region of Auvergne-Rhone Alpes. To our knowledge, this is the first report in people with HD. As observed in several other neurodegenerative disorders, an overall negative impact of the pandemic on the patients' symptoms, the delivery of health care and the caregivers emerged from our survey.

Interestingly, participants who were infected with COVID-19 did not experience a worsening of HD symptoms directly related to the infection, but they suffered from social stigma in a quarter of cases. Indeed, almost half of the participants reported a worsening or new HD symptoms related to the global health crisis and not to the COVID-19 disease itself. This could be explained with the inadequate delivery of care and physical restrictions. Actually, the most frequent worsening was experienced in gait and balance, likely related to reduced physical activity and to reduced/impossible access to physiotherapy. Similarly, the insufficient access to speech therapists care could have caused the worsening of speech and swallowing issues. Social isolation and fear about the impact of the COVID-19 pandemic on HD, along with the concerns about the consequences on their disease management and care access, could explain the frequent worsening and the new-onset of psychiatric disorders. Impaired access to regular psychologist consultations could have further worsen psychiatric issues. The worsening of fatigue could be multifactorial, related to depression, sleep and cognitive issues. Chorea could have been aggravated by anxiety and reduced physiotherapy. Moreover, the social isolation may have negatively impacted the cognitive functions of HD patients. The clinical worsening experienced by some of the participants was significant, as a quarter of them needed a medical treatment adaptation.

Concerning patients with COVID-19 disease not experiencing worsening of HD symptoms, we may speculate that the infection was likely of mild intensity and with rapid resolution in all cases as they were successfully managed at home, so that the impact on chorea and other neuropsychiatric issues was not clinically significant.

Despite the efforts of the health systems to face the COVID-19 crisis, the vast majority of the participants had the perception of an overall inadequate management of HD, mainly related to reduced/impossible or delayed health care access, similarly to other neurologic patients communities [12]. Moreover, one third of participants judged video-consultations and phone calls with medical staff as insufficient to replace in-person visits. Indeed, the application of telemedicine in the management of HD remains limited, mainly due to cognitive and motor issues in this population.

The clinical worsening, the social isolation and the impaired access to health care systems had a negative impact also on the caregivers and families of more than half of participants, with a further worsening of these patients' management. In order to improve the management of HD population in future pandemic waves, health care services should target the needs expressed by people with HD, mainly improving the access to health services and to provide adequate advice and information.

The major limitation of our survey is the small sample of participants, coming from a limited geographic area, and likely not fully representative of the HD population. Most of them were completely independent so that the patients' population with more severe disease has been likely under-estimated. Moreover, only a small percentage (8%) of the HD asymptomatic population participated to the survey, likely because less implied in the AHF network.

In conclusion, despite its limitations, our survey highlights the negative impact of the first wave of COVID-19 pandemic on the healthcare of HD population. The COVID-19 emergency made HD patients even more fragile and vulnerable. In the future, health policies should give priority to improve the care of patients with rare diseases such as HD.

## Funding

This research did not receive any specific grant from funding agencies in the public, commercial, or not-for-profit sectors.

## CRediT authorship contribution statement

**Sara Meoni:** Writing – review & editing, Writing – original draft, Validation, Supervision, Methodology, Formal analysis, Data curation, Conceptualization. **Elena Moro:** Writing – review & editing, Validation.

## Declaration of competing interest

The Authors reported no financial disclosures.
